# Human embryonic stem cell-derived cardiomyocyte therapy in mouse permanent ischemia and ischemia-reperfusion models

**DOI:** 10.1186/s13287-019-1271-4

**Published:** 2019-06-13

**Authors:** You Yu, Nianci Qin, Xing-Ai Lu, Jingjing Li, Xinglong Han, Xuan Ni, Lingqun Ye, Zhenya Shen, Weiqian Chen, Zhen-Ao Zhao, Wei Lei, Shijun Hu

**Affiliations:** 1 Department of Cardiovascular Surgery of the First Affiliated Hospital & Institute for Cardiovascular Science, State Key Laboratory of Radiation Medicine and Protection, Medical College, Soochow University, Suzhou, 215000 China; 2 Institute of Microcirculation & Department of Pathophysiology of Basic Medical College, Hebei North University, Zhangjiakou, 075000 Hebei China

**Keywords:** Ischemic heart disease, Embryonic stem cell, Cardiomyocyte, Cell therapy

## Abstract

**Background:**

Ischemic heart diseases are still a threat to human health. Human pluripotent stem cell-based transplantation exhibits great promise in cardiovascular disease therapy, including heart ischemia. The purpose of this study was to compare the efficacy of human embryonic stem cell-derived cardiomyocyte (ESC-CM) therapy in two heart ischemia models, namely, permanent ischemia (PI) and myocardial ischemia reperfusion (IR).

**Methods:**

Human embryonic stem cell-derived cardiomyocytes were differentiated from engineered human embryonic stem cells (ESC-Rep) carrying green fluorescent protein (GFP), herpes simplex virus-1 thymidine kinase (HSVtk), and firefly luciferase (Fluc). Two different heart ischemia models were generated by the ligation of the left anterior descending artery (LAD), and ESC-Rep-derived cardiomyocytes (ESC-Rep-CMs) were transplanted into the mouse hearts. Cardiac function was analyzed to evaluate the outcomes of ESC-Rep-CM transplantation. Bioluminescence signal analysis was performed to assess the cell engraftment. Finally, the inflammation response was analyzed by real-time PCR and ELISA.

**Results:**

Cardiac function was significantly improved in the PI group with ESC-Rep-CM injection compared to the PBS-injected control, as indicated by increased left ventricular ejection fraction (LVEF) and left ventricular fractional shortening (LVFS), as well as reduced fibrotic area. However, minimal improvement by ESC-Rep-CM injection was detected in the IR mouse model. We observed similar engraftment efficiency between PI and IR groups after ESC-Rep-CM injection. However, the restricted inflammation was observed after the injection of ESC-Rep-CMs in the PI group, but not in the IR group. Transplantation of ESC-Rep-CMs can partially preserve the heart function via regulating the inflammation response in the PI model, while little improvement of cardiac function in the IR model may be due to the less dynamic inflammation response by the mild heart damage.

**Conclusions:**

Our findings identified the anti-inflammatory effect of ESC-CMs as a possible therapeutic mechanism to improve cardiac function in the ischemic heart.

**Electronic supplementary material:**

The online version of this article (10.1186/s13287-019-1271-4) contains supplementary material, which is available to authorized users.

## Background

Ischemic heart disease is a leading cause of death worldwide [1]. Cardiac ischemia, known as myocardial infarction (MI), usually triggers massive cardiomyocyte death. Due to the limited regenerative capacity of the heart [2], most patients who suffered from permanent ischemia (PI) will develop into heart failure. Currently, reperfusion strategies, such as thrombolytic therapy or primary percutaneous coronary intervention, are still the standard and most effective therapeutic treatment for acute myocardial infarction [3]. Despite the potential to salvage myocardial ischemia, ischemia reperfusion (IR) usually leads to paradoxical cardiomyocyte dysfunction and worsens heart damage in what is known as ischemia-reperfusion injury [4, 5]. In contrast to IR, permanent ischemia significantly changes heart structure and function [6]. It remains uncertain whether transplantation outcomes are correlated with the ischemic microenvironment in the heart. Thus, preclinical studies in animal models of heart ischemia are necessary to fully evaluate the cell therapeutic efficacy.

Over the past decades, extensive efforts have been made to repair heart tissue by promoting cardiac regeneration [7, 8], through inducing cardiomyocyte proliferation or reprogramming fibroblasts into cardiomyocytes [9–11]. Nevertheless, the limited cardiomyocyte proliferation rate and lower direct reprogramming efficiency restrict their application in heart repair [12]. In recent years, pluripotent stem cell-based regenerative therapy has shown great promise in heart repair and functional improvement [13]. Among several cell types derived from human pluripotent stem cells, cardiomyocytes hold great promise for cardiac repair, and human embryonic stem cell-derived cardiomyocytes (ESC-CMs) can improve cardiac function and attenuate myocardial remodeling after myocardial infarction [14–17]. However, the efficacy of ESC-CM-mediated cardiac repair is still controversial. We speculated that different ischemia models may result in different heart repair and cell retention outcomes.

Embryonic stem cell-derived cardiomyocytes exhibited great application perspective in heart disease therapy, while the efficiency of cell transplantation is still low, and the underlying mechanisms need to be further investigated [13]. Early reperfusion after ischemia benefits heart function in the clinic [18]. However, whether this operation could improve cell transplantation efficiency was still unknown. The animal PI and IR models are widely used in heart repair studies [19]. To provide evidence for optimizing cell transplantation strategy, this study investigated the efficacy of ESC-CM therapy in the two different heart ischemia models. Meanwhile, we explored the possible reasons for distinct cardiac repair outcomes of ESC-CMs in these two models.

## Methods

### Human embryonic stem cell culture

For routine maintenance, undifferentiated ESCs were cultured on growth factor-reduced Matrigel (Corning, USA)-precoated dishes in complete mTeSR™1 medium (STEMCELL Technologies, Canada) and passaged every ~ 4 days using 0.5 mM EDTA (Sigma, USA). Rho-associated protein kinase (ROCK) inhibitor thiazovivin (Selleck Chemicals, USA) was added during cell passaging to prevent dissociation-induced ESC apoptosis.

### Generation of reporter-engineered human embryonic stem cells (ESC-Rep)

The DNA fragment containing green fluorescent protein (GFP), Herpes Simplex Virus-1 Thymidine Kinase (HSVtk), and firefly luciferase (Fluc) was inserted to AAVS1 locus of *PPP1R12C* gene through CRISPR/Cas9-mediated homologous recombination. The donor plasmid contains CAG promoter-derived three reporter genes using 2A peptide fusion method for co-expression. A splice acceptor element and a 2A linker were placed in front of the puromycin-polyA cassette, which expressed the puromycin resistance gene for positive clone selection. After transfection, puromycin-resistant and GFP^+^ cells were enriched by puromycin (1 μg/mL), and single GFP^+^ cell clone was picked for expansion. Finally, the expression of three reporter genes and pluripotency markers were confirmed. The edited human embryonic stem cell line was named as ESC-Rep.

### Cardiomyocyte differentiation

ESCs were split and cultured as described above. When cells reached ~ 90% confluence, cardiomyocyte differentiation was initiated by changing the culture medium to differentiation medium CDM3 [20]. During cardiomyocyte differentiation, cells were treated with 5 μM of the glycogen synthase kinase 3-β inhibitor CHIR99021 (Sigma, USA) on days 0–2 and 2 μM of the Wnt pathway inhibitor Wnt-C59 (Selleck Chemicals, USA) on days 4–6. The medium was changed daily, and spontaneous beating was noted from day 9. For cardiomyocyte purification, the cells were replated and cultured in CDM3L medium, which consisted of glucose-free RPMI 1640 (Thermo Fisher, USA) and 5 mM sodium dl-lactate (Sigma, USA). Cardiomyocytes were then split at 1:4 with 0.25% trypsin (Sigma, USA) containing 0.1 mM EDTA and seeded on 0.1% gelatin-coated dishes in CDM3.

### Animal studies

Because female mice have less pronounced maladaptive remodeling and higher survival rate after heart injury [21], female severe combined immune deficiency (SCID/beige) mice (8 weeks old) were used in this study to exclude the gender influence on the heart function. Mice were randomly grouped into 5 groups: Sham control (*n* = 15), PI groups that received permanent coronary occlusion followed by PBS (*n* = 15) or ESC-Rep-CM injection (*n* = 18), and IR groups subjected to transient ischemia for 30 min followed by reperfusion and injection of PBS (*n* = 15) or ESC-Rep-CMs (*n* = 18). After left thoracotomy, the left anterior descending artery (LAD) was ligated, either permanently (PI groups) or temporarily (IR groups), with a 7–0 prolene suture 1 mm caudally from the tip of the left auricle. In the IR group, the LAD was transiently occluded for 30 min before removing the suture. After the PI or IR operation, a total of 1 × 10^6^ ESC-Rep-CMs in 9 μL PBS were injected in equal portions into three sites beneath the ligation position. In the PBS group, an equal volume of PBS was injected at the same positions. To evaluate the influence of inflammatory microenvironment in the functional improvement of IR hearts, the mice were randomized into 5 groups (*n* = 5 for each group) including Sham control, PBS, ESC-Rep-CMs, IL-10, and ESC-Rep-CMs+IL-10. The mouse recombinant IL-10 (50 μg/kg) was subcutaneously injected into the mice in IL-10 and ESC-Rep-CMs+IL-10 groups at days 0, 1, and 3 post-surgery.

The Visual Sonics Vevo 2100 system equipped with a medium-frequency (30 MHz) MS-400 transducer was used for evaluating the cardiac function as previously reported [22]. Generally, two-dimensional long-axis and short-axis left ventricle imaging were collected for analysis. M-mode tracings were recorded through the septum and posterior LV walls to measure LV dimension and wall thickness. Left ventricular end-diastolic diameter and end-systolic diameter were measured and used to calculate left ventricular ejection fraction (LVEF) and fraction shorting (LVFS). Pulse-wave Doppler was recorded from the apical 4-chamber view. *E* (the peak early transmitral flow velocity), *A* (the peak late transmitral flow velocity), and *E*/*A* (the ratio of the peak early transmitral flow velocity to the peak late transmitral flow velocity) were measured and analyzed as previously described [23]. In addition to the cardiac functional evaluation by echocardiography, the heart fibrosis area and fibrotic marker expression were also detected to determine the injury degree of heart.

### Quantitative real-time PCR

To analyze mRNA expression, cells were dissociated, and pellets of cells were snap-frozen in liquid nitrogen and stored at − 80 °C. Total RNA was isolated using Trizol Reagent (Sigma, USA), and cDNA was produced using a PrimeScript™ 1st Strand cDNA Synthesis Kit (Clontech, USA). Real-time PCR was performed using PrimeScript™ RT Master Mix (Clontech, USA) in a StepOnePlus™ Real-Time PCR System (Thermo Fisher, USA) as previously reported [24]. Gene expression levels were normalized to the endogenous reference gene *GAPDH* and assessed using the comparative threshold cycle (2^−ΔΔCt^) method. All primer sequences are listed in Additional file 1: Table S1.

### Immunofluorescence

Cells and heart sections were fixed in 4% paraformaldehyde (PFA) for 10 min at room temperature (RT). After permeabilized with 0.2% Triton X-100 in PBS, samples were blocked in 5% BSA (Sigma, USA) at RT for 1 h, then incubated with indicated primary antibodies at 4 °C overnight. After washing with PBS-T (0.1% Tween 20 in PBS), cells or sections were incubated with corresponding fluorescent secondary antibodies at RT for 1 h, and then counterstained with Hoechst 33342 (Sigma, USA). Images were captured with a confocal microscope (ZEISS, Germany). Antibodies used in this study are listed in Additional file 1: Table S2.

### Bioluminescence imaging in vitro and in vivo

For in vitro imaging, cells were seeded as indicated. Before analysis, cells were washed with D-PBS carefully and incubated in medium containing 150 μg/mL d-luciferin (Gold Biotechnology, USA) at 37 °C for 5 min. Signals were detected by an in vivo imaging system (PerkinElmer, USA). For in vivo imaging, mice were anesthetized and simultaneously received an intraperitoneal injection of d-luciferin at 150 mg/kg (luciferin/body weight). Ten minutes post-injection, images were recorded for 10 min with 1-min acquisition intervals. As previously described, the bioluminescence from a fixed region of interest was processed with IVIS imaging systems, Living Image 4.5 (PerkinElmer, USA), and the data for cell retention and survival were quantified in units of photons per second per centimeter squared per steradian (p/s/cm^2^/sr) [25].

### Histological examination

Hearts from PI and IR groups at 4 weeks after surgery were harvested, fixed, dehydrated, and immersed in O.C.T. compound. Serial 5-μm-thick frozen sections were collected following a standard protocol. To determine the fibrotic degree of the heart, sections were stained with Masson’s trichrome (MT) using the Trichrome Stain kit (Sigma, USA) according to the manufacturer’s instructions. Collagen deposition was visualized as blue staining. Immunofluorescence staining for human nuclear antigen (HNA) was used to locate ESC-Rep-CM engraftment.

### ELISA assay

On days 1, 3, and 7 after surgery, blood was obtained via retroorbital bleeding and allowed to clot at RT. The levels of inflammation factors in sera, namely, TNF-α, IL-6, and IL-10, were measured using the Mouse TNF alpha ELISA kit (Abcam, USA), Mouse IL-6 ELISA kit (Abcam, USA), and Mouse IL-10 ELISA kit (Abcam, USA) as per the manufacturer’s instructions.

### Determination of MPO activity

MPO activity was measured according to the instructions of a MPO kit (Jiancheng Bio, China). Briefly, the homogenized tissue samples were sonicated to release the MPO from the tissue into the supernatant. After the addition of *o*-dianisidine hydrochloride and hydrogen peroxide, MPO activity was detected at 460 nm according to the spectrophotometer method.

### Statistical analysis

Comparisons between two groups were analyzed using Student’s *t* test. Comparisons in multiple groups were analyzed with one-way analysis of variance (ANOVA) or two-way repeated-measures analysis of variance with the Bonferroni post hoc test. Statistical significance was denoted by a *p* value of less than 0.05. Data are presented as the mean ± SEM.

## Results

### Generation and identification of ESC-derived cardiomyocytes

**Fig. 1:**
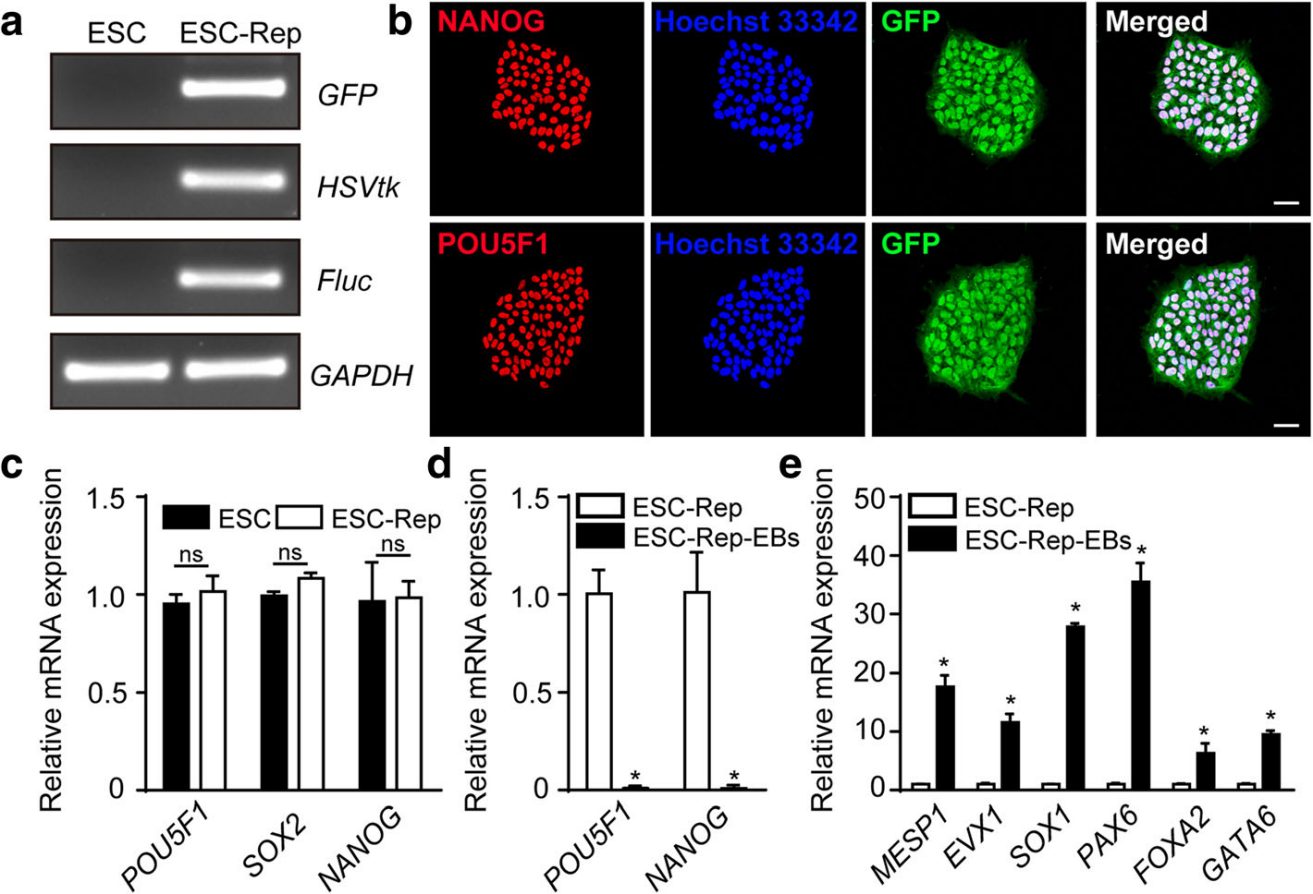
Characterization of the human embryonic stem cell reporter line ESC-Rep. **a** Identifying the DNA sequences of the three reporter genes *GFP*, *HSVtk*, and *Fluc* in ESC-Rep by semiquantitative PCR. *GAPDH* was used as a reference. **b** Immunofluorescence staining of ESC-Rep with pluripotency markers (*NANOG* and *POU5F1*). Nucleus is marked by Hoechst 33342 (blue). Scale bar, 50 μm. **c** Real-time PCR analysis of genes associated with pluripotency in ESCs and ESC-Rep cells. **d** Real-time PCR analysis of pluripotency markers (*POU5F1* and *NANOG*) in ESC-Rep and differentiating EBs produced from ESC-Rep (ESC-Rep-EBs). **e** Real-time PCR analysis of germ layer markers in undifferentiated ESC-Rep and ESC-Rep differentiated EBs (ESC-Rep-EBs) (*MESP1* and *EVX1* for mesoderm, *SOX1* and *PAX6* for ectoderm, *FOXA2* and *GATA6* for endoderm). All data are presented as the mean ± SEM; Student’s *t* test; *p < 0.05, and ns, not significant

Three reporter genes, namely, green fluorescent protein (GFP), Herpes Simplex Virus-1 Thymidine Kinase (HSVtk), and firefly luciferase (Fluc), were successfully engineered into human embryonic stem cells (ESC), and the engineered cells were named as ESC-Rep (Additional file 1: Figure S1). We first confirmed the expression of all three reporter genes, as well as pluripotency markers *NANOG* and *POU5F1* (also known as *OCT4*), in ESC-Rep cells by semiquantitative PCR and immunofluorescence staining, respectively (Fig. 1a, b). The expression levels of pluripotency genes such as *POU5F1*, *SOX2,* and *NANOG* in ESC-Rep cells were comparable to those in unedited ESCs (Fig. 1c). Next, spontaneous differentiation was conducted through embryoid body (EB) formation to evaluate the pluripotency of ESC-Rep cells. Upon EB formation, the differentiated cells showed the significantly decreased expression of pluripotency markers *NANOG* and *POU5F1* (Fig. 1d), along with the highly increased expression of germ layer-specific markers (*MESP1* and *EVX1* for mesoderm, *SOX1* and *PAX6* for ectoderm, *FOXA2* and *GATA6* for endoderm) (Fig. 1e). These data indicated the ESC-Rep cell line retained self-renewal ability and pluripotency. Thus, the ESC-Rep cell line was used in the following experiments.

**Fig. 2:**
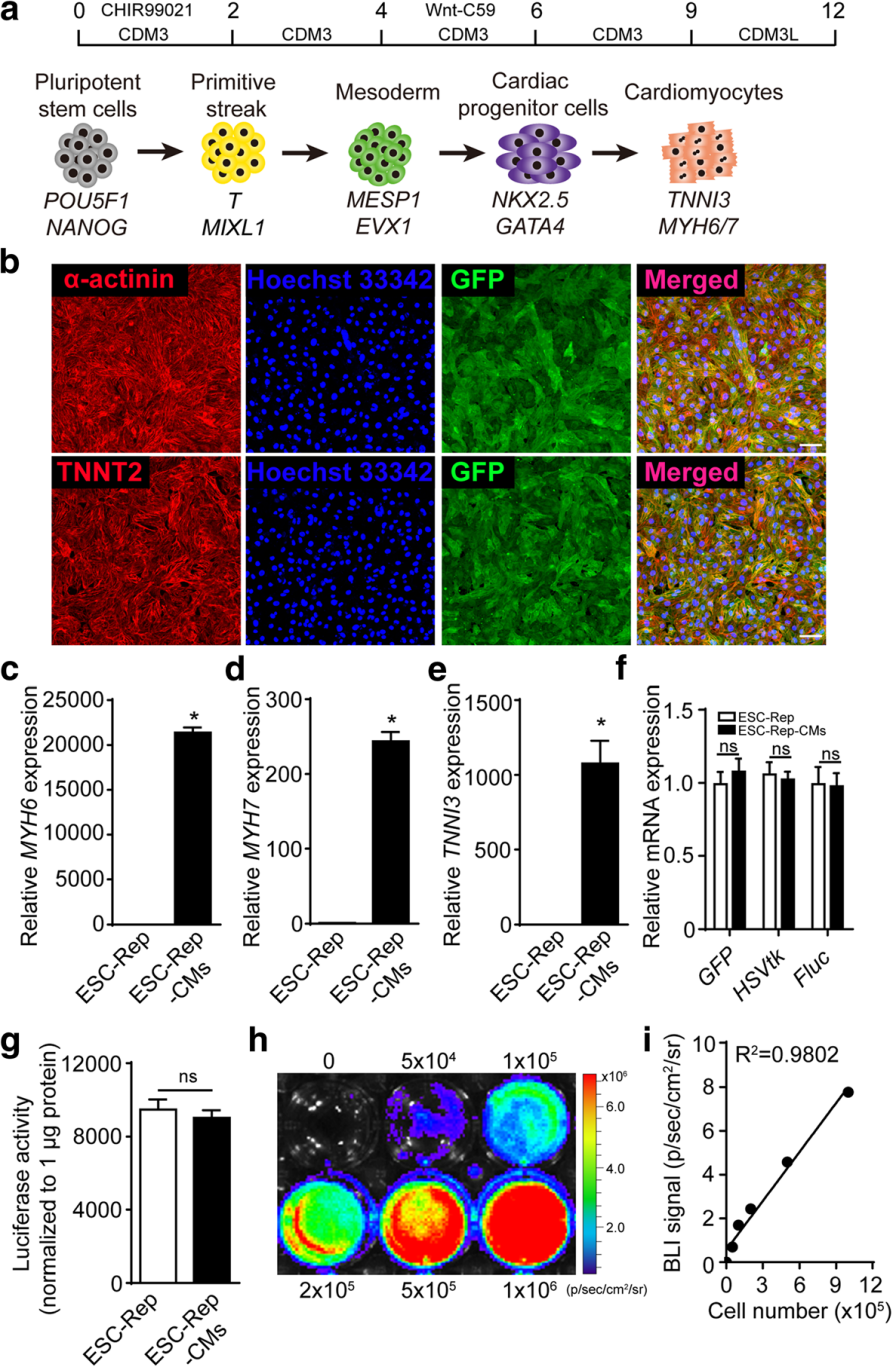
Generation of cardiomyocytes from ESC-Rep. **a** Illustration of cardiomyocyte differentiation protocol of ESCs and stage-specific cell fate as well as representative markers. **b** Immunofluorescence staining of ESC-Rep-CMs for cardiac structural markers alpha-actinin and troponin T (TNNT2). Nucleus is marked by Hoechst 33342 (blue). Scale bar, 50 μm. Real-time PCR analysis of cardiac-specific gene expression in ESC-Rep-CMs, including *MYH6* (**c**), *MYH7* (**d**), and *TNNI3* (**e**). **f** Real-time PCR analysis of the three reporter genes (*GFP*, *HSVtk*, and *Fluc*) in ESC-Rep and ESC-Rep-CMs. **g** Luciferase activity measurement of ESC-Rep and ESC-Rep-CMs. The luciferase activity was normalized to 1 μg protein. **h** Luciferase activity in ESC-Rep-CMs in 6-well plates. **i** A robust correlation existed between cell number and bioluminescence signals (*R*^2^ = 0.98). All data are presented as the mean ± SEM; Student’s *t* test; *p < 0.05, and ns, not significant


Cardiomyocyte differentiation was performed by a stepwise protocol as previously described with some modification [24] (Fig. 2a). The expression of stage-specific markers showed the successful differentiation of cardiomyocytes (Additional file 1: Figure S2). After metabolic purification in the medium containing lactate and without glucose [20, 24], we obtained about 98% cardiac muscle troponin T (TNNT2)-positive cells (Additional file 1: Figure S3A) and these enriched ESC-Rep-derived cardiomyocytes (ESC-Rep-CMs) expressed cardiac-specific markers including sarcomeric α-actinin, TNNT2, and GFP (Fig. 2b). Cardiomyocyte-specific markers, such as myosin heavy chain 6 (*MYH6*), myosin heavy chain 7 (*MYH7*), and troponin I3 (*TNNI3*), were significantly expressed in ESC-Rep-CMs (Fig. 2c–e), and cardiomyocyte spontaneous beating and electrophysiological characteristic were also recorded (Additional file 2: Video S1 & Additional file 1: Figure S3B). These data indicated we successfully obtained the functional cardiomyocytes from ESC-Rep. The three reporter genes showed unbiased expression in ESC-Rep and ESC-Rep-CMs (Fig. 2f). Luciferase activity was not changed after cardiomyocyte differentiation from ESC-Rep cells (Fig. 2g). Next, the luciferase activity in ESC-Rep-CMs was detected by bioluminescence imaging (BLI) and showed a positive linear correlation with cell number (Fig. 2h, i). In summary, the ESC-Rep-CMs expressed three reporters and could be traced by molecular imaging in vitro, providing a useful tool for in vivo imaging.

Additional file 2:
**Video S1.** The representative movie of spontaneous beating cardiomyocytes derived from reporter cells at day 20 after purification. (MP4 848 kb)


### Cardiac performance after ESC-Rep-CM injection in two heart ischemia models

**Fig. 3:**
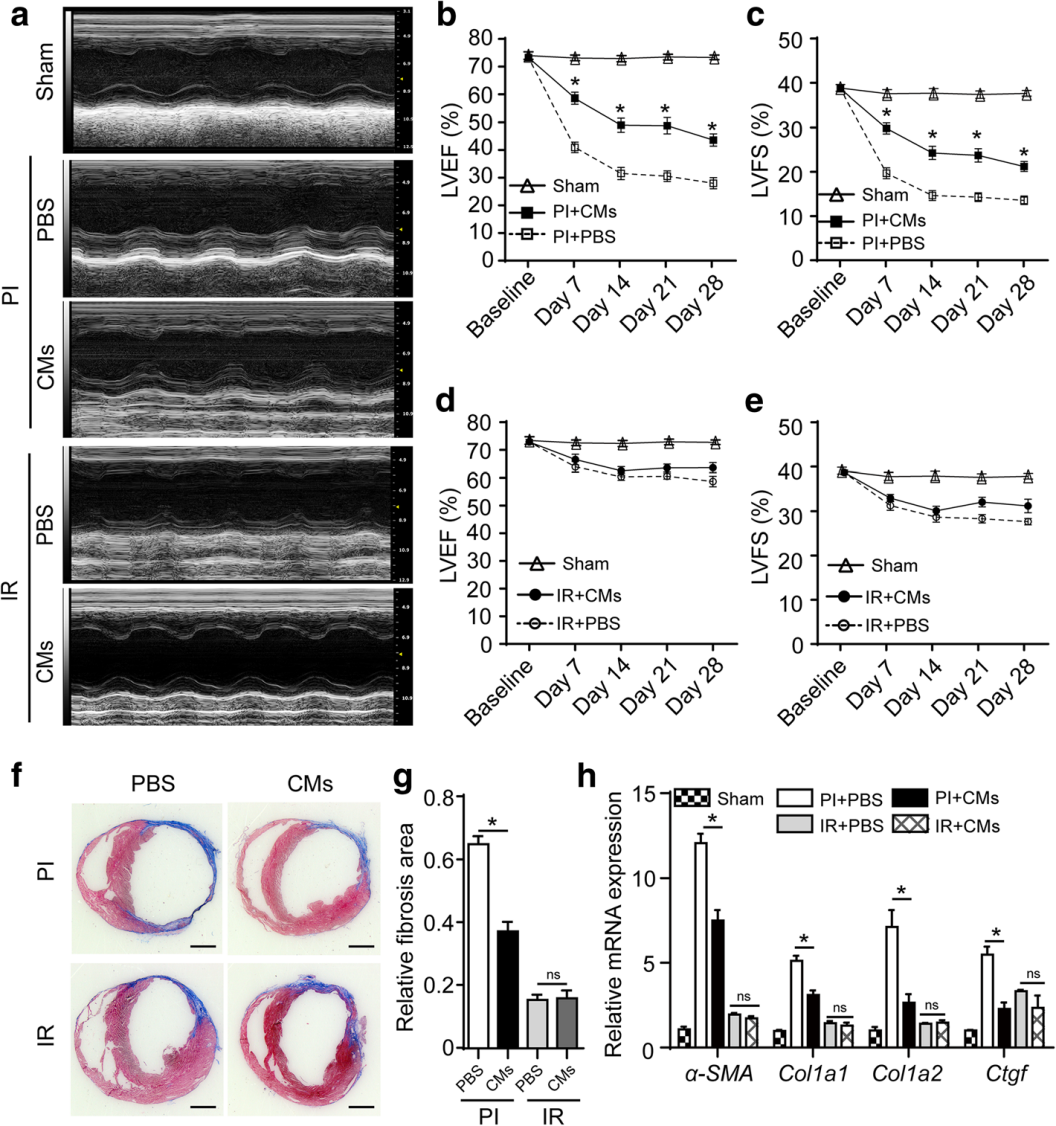
ESC-Rep-CM injection in two heart ischemia models. **a** Representative echocardiogram of mice at day 28 in the Sham control, PI group after PBS or ESC-Rep-CM injection, and the IR group after the injection of PBS or ESC-Rep-CMs. *n* = 6 per group. **b** Quantitative analysis of left ventricular ejection fraction (LVEF) in PBS or ESC-Rep-CM-injected PI mice from day − 1 (baseline) to day 28. **c** Quantitative analysis of left ventricular fractional shortening (LVFS) in PBS or ESC-Rep-CM-injected PI mice from day − 1 to day 28. **d** Quantitative analysis of LVEF in the IR groups from day − 1 to day 28. **e** Quantitative analysis of LVFS in the IR groups from day − 1 to day 28. **f** Representative Masson trichrome staining images of PI and IR hearts at day 28 after PBS or ESC-Rep-CM injection. Scale bar, 1 mm. *n* = 3 per group. **g** Statistical analysis of relative fibrosis area from Masson trichrome staining data. *n* = 3 per group. **h** Real-time PCR analysis of heart tissue for fibrosis markers in the Sham control, PI group treated with PBS or ESC-Rep-CMs, and IR groups treated with PBS or ESC-Rep-CMs at day 28. *n* = 3 per group. All data are presented as the mean ± SEM; one-way ANOVA or two-way repeated-measures ANOVA; * *p* < 0.05, and ns, not significant

SCID/beige mice were used to establish the PI model by permanent coronary artery ligation and the IR model by reperfusion after 30-min transient ischemia. Ischemia was confirmed by bleaching the myocardium. Reperfusion was started by releasing the suture and removing the tube, and a typical hyperemia of injured myocardium in the first few minutes was observed (Additional files 2, 3, 4, and 5: Video S2-S4). Myocardial ischemia was further proved by the identified ST-segment elevation in electrocardiograph (ECG) after coronary artery ligation (Additional file 1: Figure S4). In cell injection groups, 10^6^ ESC-Rep-CMs were injected into three sites beneath the ligation position per mouse. Left ventricular function was evaluated by echocardiography in both models after PBS or ESC-Rep-CM injection. The representative images of M-mode were shown in Fig. 3a and Additional file 1: Figure S5. In the PI model, mice that received ESC-Rep-CM injection displayed dramatic improvement of systolic heart function, as indicated by the significant increases in LVEF and LVFS on days 7–28 when compared to PBS-injected mice (Fig. 3b, c). However, minimal differences in both LVEF and LVFS were observed between PBS- and ESC-Rep-CM-injected IR mice (Fig. 3d, e). Next, the infarct area in all groups was analyzed by Masson’s trichrome staining. A significantly larger infarct area was observed in the PI group when compared with the IR group. The relative area of infarction was smaller in ESC-Rep-CM-injected PI hearts than that in PBS-treated control (Fig. 3f, g). These results were also confirmed by the expression of fibrotic markers, including alpha smooth muscle actin (*α-SMA*), connective tissue growth factor (*Ctgf*), and type I collagen (*Col1a1* and *Col1a2*) (Fig. 3h). Thus, heart function was well preserved in the PI group by ESC-Rep-CM therapy. However, only minimal improvement of cardiac function was seen in the IR group.

### Retention of the transplanted ESC-Rep-CMs in mouse heart

**Fig. 4:**
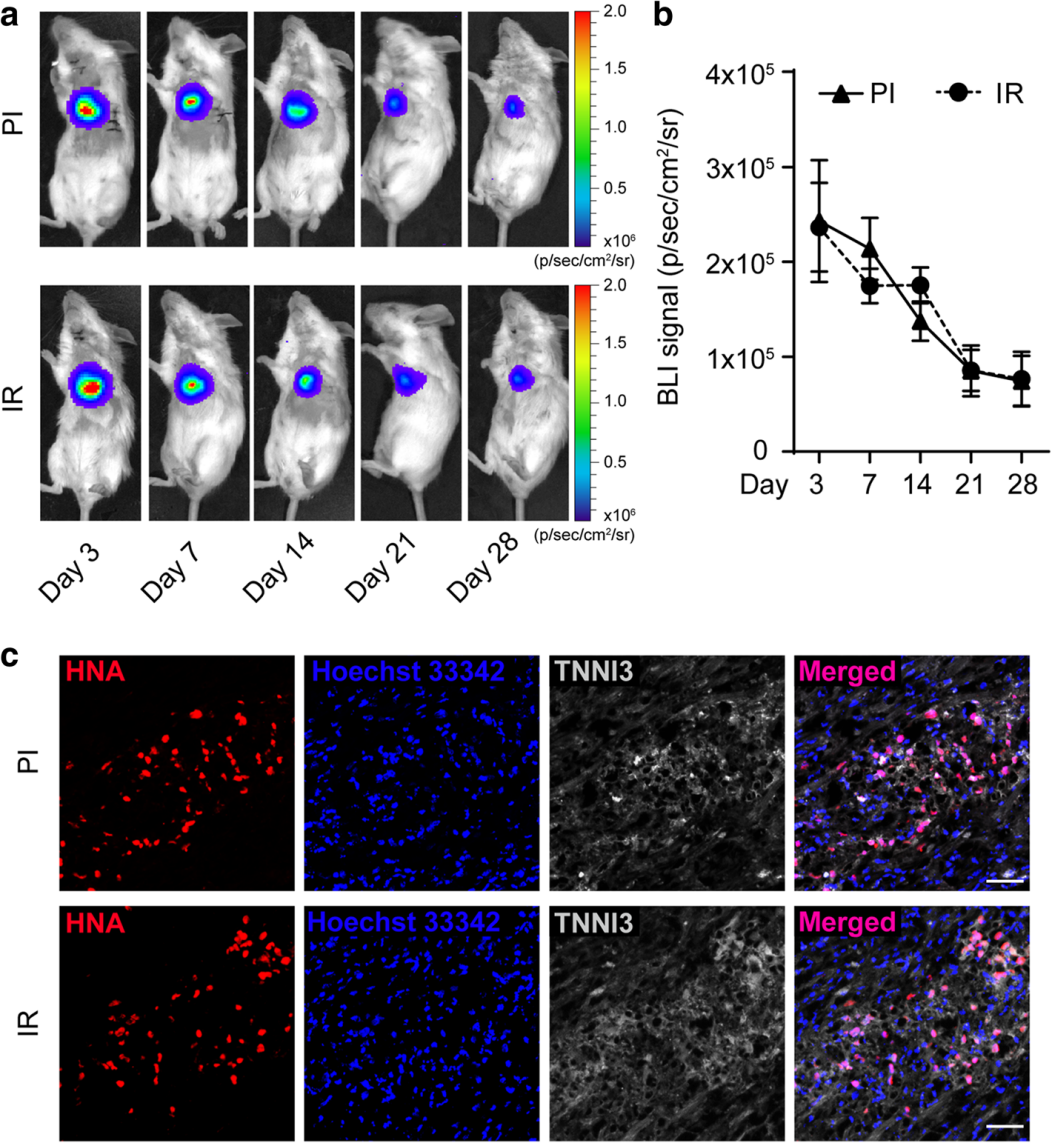
ESC-Rep-CM retention in both PI and IR hearts after cell transplantation. **a** Representative in vivo bioluminescence images of PI and IR mice on the indicated days after ESC-Rep-CM transplantation. **b** Quantitative analysis of BLI signals in the PI and IR groups. *n* = 4 for each group. **c** Immunofluorescence of heart sections from the PI and IR groups at day 7 for human nuclear antigen (HNA, red), cardiac troponin I (TNNI3, gray), and cell nuclei (Hoechst 33342, blue). *n* = 3 per group. Scale bar, 50 μm. All data are presented as the mean ± SEM

Since mild cardiac function improvement was observed in the IR group after ESC-Rep-CM therapy, we speculated whether the transplanted ESC-Rep-CMs failed to reside in IR hearts. We therefore analyzed the in vivo BLI signals in both PI and IR groups after cell injection. Surprisingly, the collected data revealed ESC-Rep-CMs were equally engrafted in both groups, although the BLI signals were attenuated as the experimental period extended. Interestingly, no significant difference in BLI signals was observed between the PI and IR groups, indicating comparable cell retention in the two disease models (Fig. 4a, b). Double staining of human nuclear antigen (HNA)/TNNI3 and HNA/GFP were performed to distinguish human ESC-Rep-CMs from mouse cells in vivo, and the results showed similar transplanted cell engraftment in mouse hearts in both PI and IR groups at day 7 (Fig. 4c and Additional file 1: Figure S6).

### Inflammation response in mouse heart after ESC-Rep-CM transplantation

**Fig. 5:**
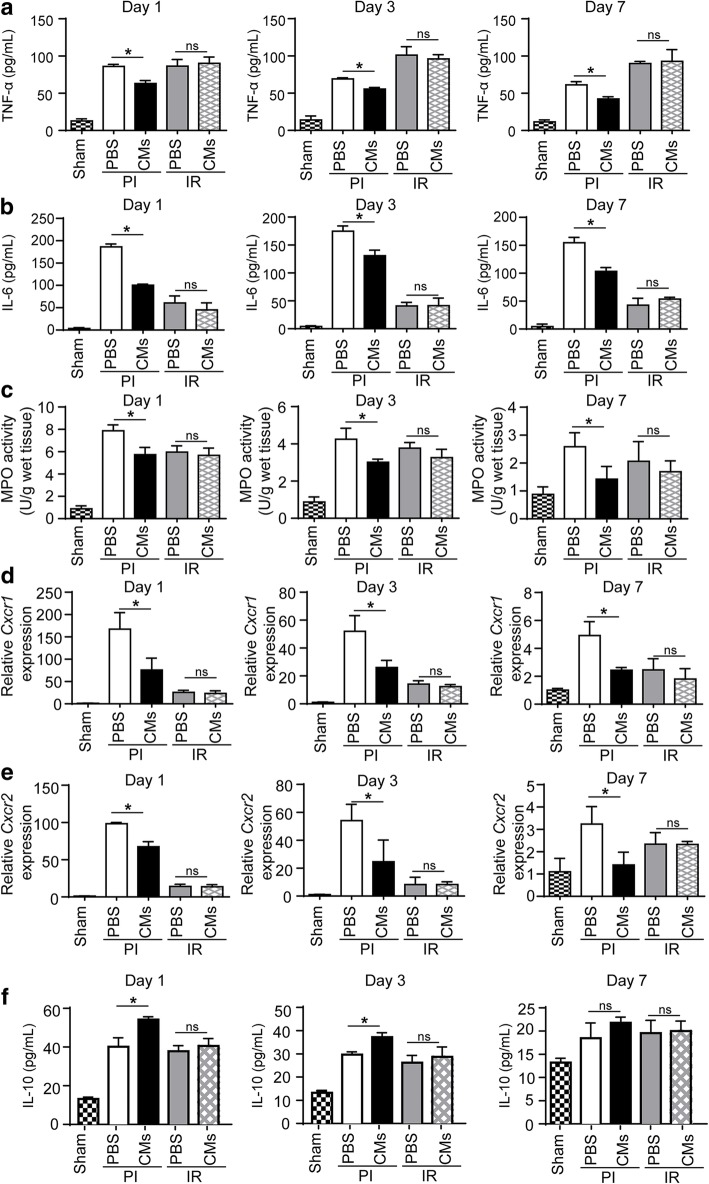
Inflammatory response in the PI and IR mice after cell transplantation. ELISA of TNF-α (**a**) and IL-6 (**b**) in mouse sera from the Sham group, PI groups treated with PBS or ESC-Rep-CMs, and IR groups treated with PBS or ESC-Rep-CMs, at day 1, day 3, and day 7 after cell transplantation. *n* = 3 per group. **c** Myeloperoxidase analysis (MPO) of left ventricles from different groups. *n* = 3 per group. Real-time PCR analysis of left ventricles for neutrophils surface receptor *Cxcr1* (**d**) and *Cxcr2* (**e**) expression. **f** ELISA of IL-10 in mouse sera from the Sham group, PI groups treated with PBS or ESC-Rep-CMs, and IR groups treated with PBS or ESC-Rep-CMs at day 1, day 3 and day 7 after cell transplantation.* n* = 3 per group. All data are presented as the mean ± SEM; one-way ANOVA; *p < 0.05, and ns, not significant

Inflammation response is an important process in cardiac remodeling after myocardial infarction [26]. Thus, we wondered whether the inflammation response was involved in ESC-Rep-CM therapy. In our two models, the inflammatory microenvironment, including interleukin levels and MPO activity, was less serious in the IR model than that in the PI model (Fig. 5), which was accordant with the previous reports [6, 27, 28]. As measured by ELISA on serum samples, the levels of inflammatory factors, such as tumor necrosis factor alpha (TNF-α) and interleukin 6 (IL-6), were dramatically elevated in both PI group and IR group compared with the Sham group. After ESC-Rep-CM transplantation in the PI group, the levels of both TNF-α and IL-6 were markedly reduced compared with the PBS group. This inhibition of serum inflammatory factors was not observed in the IR group, although it received ESC-Rep-CMs (Fig. 5a, b). The mRNA expression of *Tnf-α* and *Il-6* was also detected, showing the same trends (Additional file 1: Figure S7A & S7B). To test whether the reduced inflammation response was caused by decreased neutrophil recruitment to the heart, myeloperoxidase (MPO) activity was measured which was positively correlated with neutrophil accumulation. MPO activity was much lower in the IR group compared with the PI group, and the decreased MPO activity was observed in the PI group treated with ESC-Rep-CMs, indicating the reduced neutrophil recruitment occurred after cell injection (Fig. 5c). The neutrophil chemokine receptors *Cxcr1* and *Cxcr2* were also detected. Correspondingly, the dramatically declined expression of *Cxcr1* and *Cxcr2* was observed in the PI group treated with ESC-Rep-CMs when compared with PBS treatment. Unfortunately, although a mild upregulation of *Cxcr1* and *Cxcr2* expression was seen in the IR group when compared with the Sham group, no significant difference was observed between PBS and ESC-Rep-CM treatment (Fig. 5d, e). As reported, IL-10 functions as an anti-inflammatory cytokine to preserve heart function after heart ischemia [29], so we detected IL-10 in our models. After ESC-Rep-CM transplantation, IL-10 protein and mRNA were significantly increased in the PI group, rather than the IR group (Fig. 5f and Additional file 1: Figure S7C).

**Fig. 6:**
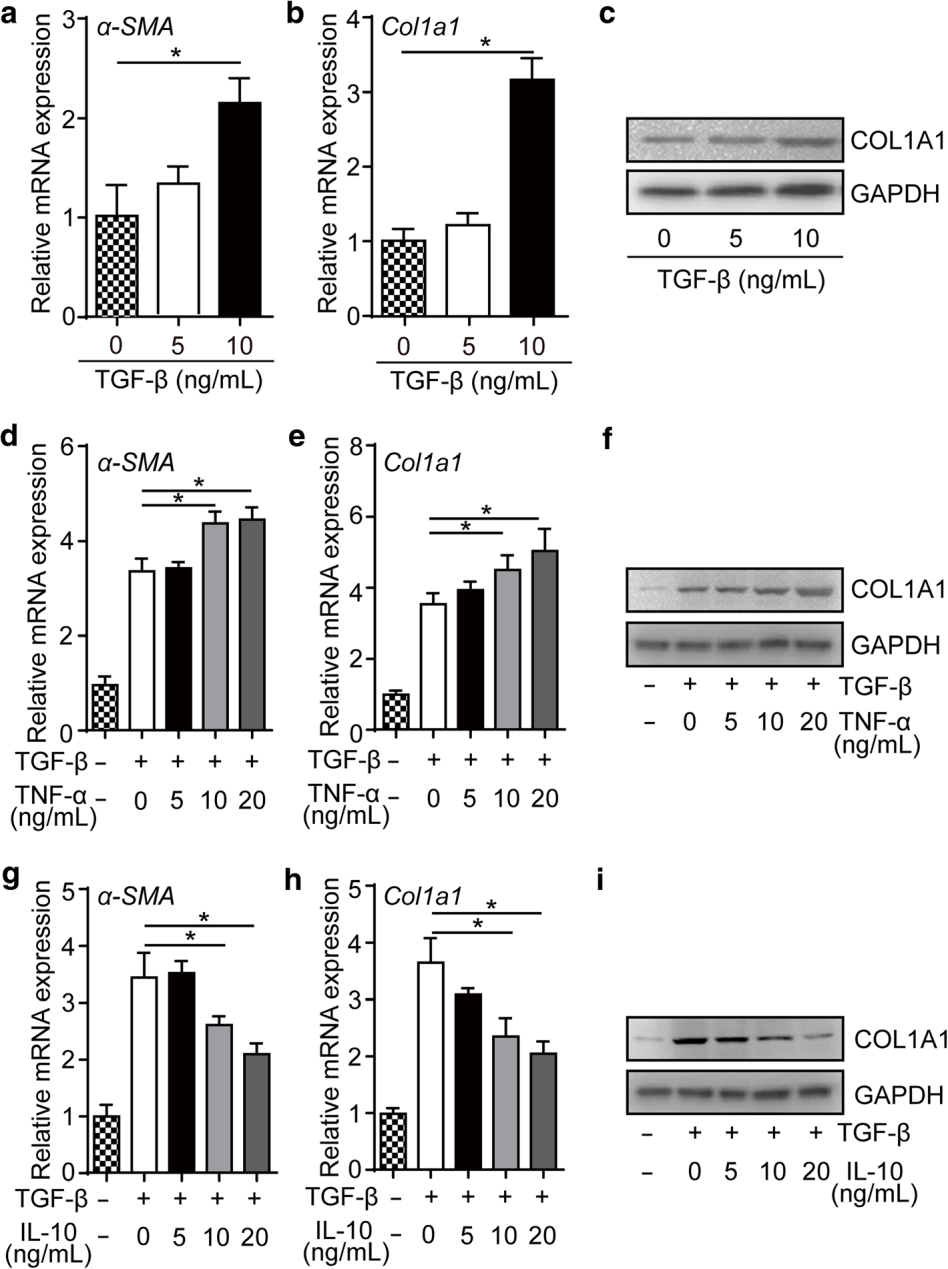
Inflammatory factors regulate fibrosis in vitro. Real-time PCR analysis of *α-SMA* (**a**) and *Col1a1* (**b**) in mouse fibroblasts after TGF-β treatment. **c** The protein level detection of COL1A1 after TGF-β treatment by western blot. Real-time PCR analysis of *α-SMA* (**d**) and *Col1a1* (**e**) mRNA expression after TGF-β and TNF-α treatment. **f** Western blot showed COL1A1 protein expression level after TGF-β and TNF-α treatment. Real-time PCR analysis of *α-SMA* (**g**) and *Col1a1* (**h**) after TGF-β and IL-10 treatment. **i** Western blot showed COL1A1 after TGF-β and IL-10 treatment. All data are presented as the mean ± SEM; one-way ANOVA; *p < 0.05, and ns, not significant

Since the inflammatory response and infarct area were reduced after ESC-Rep-CM transplantation in the PI model, we further tested the effect of inflammatory factors on fibrosis in vitro. Mouse fibroblasts were treated with transforming growth factor-β (TGF-β) for 48 h, and successful fibrotic induction was identified by significant upregulation of fibrosis markers α-SMA and COL1A1 (Fig. 6a–c). Compared to TGF-β stimulation alone, combined treatment of TGF-β and the pro-inflammatory factor TNF-α dramatically accelerated the mRNA expression of *α-SMA* (Fig. 6d) and *Col1a1* (Fig. 6e) as well as protein level of COL1A1 (Fig. 6f), while anti-inflammatory cytokine interleukin 10 (IL-10) attenuated TGF-β-induced fibrosis (Fig. 6g–i). To test whether the inhibition of the inflammatory environment could also benefit the heart function in our IR model, the mice were injected subcutaneously with mouse recombinant IL-10 at days 0, 1, and 3 post PBS or ESC-Rep-CM injection, and the mouse heart function was mildly preserved by IL-10 injection (Additional file 1: Figure S8), which was similar with previous reports on the PI model [29, 30]. Thus, these data suggested the improvement of cardiac function in PI mice might be associated with the attenuated inflammatory environment induced by ESC-Rep-CM transplantation.

## Discussion

Myocardial infarction animal models are typically induced by occlusion of the left anterior descending artery. According to the ligating time, there are two different ischemia models, which are termed PI and IR respectively in this study. Ligation for 30–45 min in an ischemia-reperfusion model is well accepted [6], but longer ischemia (> 50 min) results in unacceptably high mortality (nearly 100%) either during the ischemic process or in the first week after the surgery [27]. Thus, a conservative ligating time (30 min) was used in the ischemia-reperfusion model (IR) in our study. Our data on infarct area differences between the PI and IR groups were consistent with other studies, which also observed a significantly larger infarct area after permanent ischemia [31]. The cardiac function of IR mice was also much better than that of PI mice. In this regard, our study revealed that ESC-Rep-CM transplantation could partially preserve the cardiac function of PI mice, but showed a mild benefit in the cardiac performance of IR mouse hearts, indicating the degree of injury affected the outcome of cell therapy. Considering the similar cell retention in two different models, we speculated the degree of heart injury and the different heart remodeling might affect the outcomes of cell therapy. Meanwhile, we noticed the gradual loss of transplanted cells might attenuate the efficiency of cell therapy. Thus, the strategy of cell transplantation needs to be further optimized. Previous studies have shown the combination of biomaterial and cytokines can promote cell survival and retention in the infarct area [32, 33], and these methods should further benefit the heart function in both PI and IR models. Due to the comparable characteristics between ESCs and induced pluripotent stem cells (iPSCs) [34–36], the same therapeutic effects of ESC- and iPSC-derived cardiomyocytes in these two heart ischemia models would be expected.

Transplanted cardiomyocytes can improve the heart function in several animal MI models through functional integration, paracrine-mediated inhibition of cardiomyocyte apoptosis, and enhancement of the vasculogenic response [15, 37–39]. To our knowledge, the extremely high rate of post-transplantation cell death is a major issue that limits the functional efficacy of cell therapy for ischemic heart disease. We therefore first evaluated the engraft efficiency of ESC-Rep-CMs in the two different cardiac ischemia models, but we did not find an obvious difference in cell retention. Thus, the distinct cardiac improvement of ESC-Rep-CM transplantation in the PI and IR is not due to grafting efficiency and might be related to the distinct changes to the microenvironment in the two models.

Cardiac inflammation response is an important event within the first week after ischemia. After injury, the inflammatory response localizes into the infarcted zone, and it leads to the migration of monocytes, neutrophils, and macrophages [40]. Among these, neutrophils have traditionally been regarded as pro-inflammatory effectors and are undoubtedly major effectors of acute inflammation [41]. In a mouse model, when TGF-β was specifically blocked in cardiomyocytes, cardiac function was improved due to decreased neutrophil recruitment to the heart [42]. These results indicated that the inflammatory response was very important for heart remodeling and function. Thus, if cardiac inflammation could be repressed at the right time after injury, it may be beneficial for cardiac performance. A recent study revealed a benefit of transplanted myoblasts in inhibiting the inflammatory response in cardiac tissue after MI [43]. We therefore asked whether inflammation repression was positively correlated with improved cardiac function. Based on our data, inflammation was alleviated after cardiomyocyte injection in the PI group with the decreasing levels of TNF-α and IL-6 and the increasing level of IL-10. MPO activity was also decreased. However, in the IR group, inflammatory response and MPO activity were not changed. This may have been caused by the less dynamic change in inflammation profile of the IR group. Because temporary ischemia time in the IR model was 30 min in our study, the cardiac function was still preserved, and the inflammation reaction was less severe than permanent ischemia. IL-10 is a potent anti-inflammatory cytokine as well as a cardio-protective factor in the ischemic heart [44]. Consistent with previous reports on the PI model, our data showed that, even in a lower inflammatory microenvironment, injection of the anti-inflammatory factor IL-10 could mildly benefit the cardiac performance. Taken together, when cell therapy is purposed to treat ischemic heart, the microenvironment in the heart such as inflammation response should also be taken into consideration.

## Conclusions

In conclusion, cardiomyocytes derived from ESCs could reside in mouse ischemia models, but the retention and survival of these cells were model-independent. Cardiac function was preserved in the PI group and this protection may be related to the decreased inflammation.

## Additional files

Additional file 1: **Figure S1.** Generation of reporter-engineered human embryonic stem cells (ESC-Rep). (A) Schematic diagram of CRISPR/Cas9-mediated homologous recombination in AAVS1 locus of *PPP1R12C* gene. (B) Procedure of ESC-Rep generation. **Figure S2.** Stage-specific gene expression during cardiomyocyte differentiation. Real-time PCR analysis for markers of pluripotency (*POU5F1* and *NANOG*), mesoderm (*T* and *MIXL1*), cardiac mesoderm (*MESP1* and *EVX1*), cardiac progenitors (*NKX2.5* and *GATA4*), and cardiomyocytes (*MYH6*, *MYH7*, and *TNNI3*) during the cardiac differentiation. **Figure S3.** Characteristics of ESC-Rep-CMs. (A) Flow cytometry assay for troponin T (TNNT2)-positive cardiomyocytes after purification. (B) Patch clamp for electrophysiological characteristics of ESC-Rep-CMs. **Figure S4. **Postoperative cardiac evaluation in mice. (A) Electrocardiogram analysis of mice in the Sham, PI, and IR groups after surgery. (B–D) Heart function analysis of mice in the Sham, PI, and IR groups at day 7 post surgery. All data are presented as the mean ± SEM; one-way ANOVA; *p < 0.05, **p < 0.01. **Table S1.** Primers used in this study. **Table S2.** Antibodies used in this study. **Table S3.** Cardiac parameters acquired from echocardiography at Day 28. (DOCX 12013 kb)

Additional file 3: **Video S2.** The representative movie of the heart in the Sham control group during the operation. (MP4 294 kb)

Additional file 4: **Video S3.** The representative movie of the heart in the PI group after ligation during the operation. (MP4 363 kb)

Additional file 5: **Video S4.** The representative movie of the heart in the IR group recording the process of reperfusion. (MP4 2100 kb)

## Data Availability

All data generated or analyzed during this study are included in this published article and its supplementary information files.

## Abbreviations

BLI: Bioluminescent imaging
CM: Cardiomyocyte
EB: Embryoid body
ESC: Embryonic stem cell
ESC-CM: Embryonic stem cell-derived cardiomyocyte
GFP: Green fluorescent protein
IR: Ischemia reperfusion
LAD: Left anterior descending artery
LVEF: Left ventricular ejection fraction
LVFS: Left ventricular fraction shortening
MI: Myocardial infarction
MPO: Myeloperoxidase
PI: Permanent ischemia
