# Supplementary material for: Human embryonic stem cell-derived cardiomyocyte therapy in mouse permanent ischemia and ischemia-reperfusion models
Source: Stem Cell Res Ther. 2019 Jun 13;10:167. doi: 10.1186/s13287-019-1271-4 (PMC6567449; doi:10.1186/s13287-019-1271-4)
Supplement: Supplementary file 2 — Figure S1. Generation of reporter-engineered human embryonic stem cells (ESC-Rep). (A) Schematic diagram of CRISPR/Cas9-mediated homologous recombination in AAVS1 locus of PPP1R12C gene. (B) Procedure of ESC-Rep generation. Figure S2. Stage-specific gene expression during cardiomyocyte differentiation. Real-time PCR analysis for markers of pluripotency (POU5F1 and NANOG), mesoderm (T and MIXL1), cardiac mesoderm (MESP1 and EVX1), cardiac progenitors (NKX2.5 and GATA4), and cardiomyocytes (MYH6, MYH7, and TNNI3) during the cardiac differentiation. Figure S3. Characteristics of ESC-Rep-CMs. (A) Flow cytometry assay for troponin T (TNNT2)-positive cardiomyocytes after purification. (B) Patch clamp for electrophysiological characteristics of ESC-Rep-CMs. Figure S4. Postoperative cardiac evaluation in mice. (A) Electrocardiogram analysis of mice in the Sham, PI, and IR groups after surgery. (B–D) Heart function analysis of mice in the Sham, PI, and IR groups at day 7 post surgery. All data are presented as the mean ± SEM; one-way ANOVA; *p < 0.05, **p < 0.01. Table S1. Primers used in this study. Table S2. Antibodies used in this study. Table S3. Cardiac parameters acquired from echocardiography at Day 28. (DOCX 12013 kb) [file 13287_2019_1271_MOESM1_ESM.docx]

**Additional file 1**

**Human embryonic stem cell-derived cardiomyocyte therapy in mouse permanent ischemia and ischemia-reperfusion models**

You Yu^1, *^, Nianci Qin^1, *^, Xing-Ai Lu^1, *^, Jingjing Li^1^, Xinglong Han^1^, Xuan Ni^1^, Lingqun Ye^1^, Zhenya Shen^1^, Weiqian Chen^1^, Zhen-Ao Zhao^2, #^, Wei Lei^1, #^, Shijun Hu^1, #^

^1^Department of Cardiovascular Surgery of the First Affiliated Hospital & Institute for Cardiovascular Science, State Key Laboratory of Radiation Medicine and Protection, Medical College, Soochow University, Suzhou 215000, China; ^2^Institute of Microcirculation & Department of Pathophysiology of Basic Medical College, Hebei North University, Zhangjiakou, Hebei 075000, China

* Co-first authors

**SUPPLEMENTAL METHODS**

**Luciferase assay *in vitro***

Luciferase activity was measured according to the instructions of the Dual-Luciferase^®^ Reporter Assay System (Promega, USA). Briefly, cells were harvested in passive lysis buffer after washed with D-PBS. The sample lysate was centrifuged to a pellet, and the supernatant was collected for luciferase assays. The luciferase activity was measured following a standard protocol using a microplate reader (Synergy H1, BioTek, USA). For data analysis, luciferase activity was normalized to lysate protein concentration and calculated as luciferase activity per microgram of protein.

**Flow cytometry**

ESC-Rep-CMs were dissociated into single cell with 0.25% Trypsin-EDTA and fixed in 1% paraformaldehyde for 10 minutes. Mouse monoclonal anti-cardiac troponin T (TNNT2) antibody (Thermo Fisher, USA) and Alexa Fluor 647 AffiniPure Donkey Anti-Mouse IgG (H+L) (Jackson ImmunoResearch, USA) were used for flow cytometry analysis. Data were acquired and analyzed using Guava easyGyte^TM^ 8 (EMD Millipore, Germany).

**Patch clamp**

The action potential (AP) of ESC-Rep-CMs was recorded in current-clamp mode with the whole-cell patch-clamp technique, using an Axopatch 200B amplifier. The ESC-Rep-CMs were identified by AP patterns recorded in normal external solution containing 14 mM KCl, 140 mM NaCl, 1mM MgCl_2_, 1.2Mm CaCl_2_, 10mM HEPES and 10mM D-Glusose (pH 7.35 with NaOH). The internal pipette solution contained 150 mM KCl, 4 mM NaCl, 1mM MgCl_2_, 5 mM HEPES, 5 mM EGTA and 115 mM potassium asparate (pH 7.2 with KOH).

**Electrocardiogram recording (ECG)**

Mice were anesthetized and placed in a supine position. Standard lead II surface ECGs were recorded by electrodes connected to the left and right upper limb and the left low limb. The elevation of the ST-segment (the period between the end of the QRS complex and the beginning of the T wave) was used to reveal the cardiac ischemia.

**Fibrosis induction *in vitro***

Mouse fibroblasts (NIH-3T3) were cultured in DMEM medium (DMEM, L-glutamine, 100 mg/ml streptomycin, 100 U/ml penicillin and 10% FBS). The cells were starved in 1% FBS of DMEM medium for 2 hours before being treated with transforming growth factor beta (TGF-β, 5ng/mL or 10ng/mL), tumor necrosis factor α (TNF-α, 5ng/mL, 10ng/mL, or 20ng/mL) plus TGF-β (10ng/mL), or IL-10 (5ng/mL, 10ng/mL or 20ng/mL) plus TGF-β (10ng/mL). After 48 hours, the cells were harvested to analyze the mRNA and protein expression of fibrosis markers.

**Western Blot**

The cells were lysed with RIPA lysis buffer. The proteins were separated by SDS-PAGE, and transferred onto a PVDF membrane (EMD Millipore, Germany). Each membrane was blocked with 5% nonfat dry milk in TBST (0.1% Tween 20 in TBS) at room temperature for 1 hour. The membrane was then incubated with primary antibody at 4 °C overnight. After three washes with TBST, the membranes were incubated with HRP conjugated-secondary antibodies at room temperature for 1 hour. The signals were detected using a Phototope (R)-HRP Western Blot Detection kit (Cell Signaling Technology, USA). The antibodies used for western blotting are listed in Supplementary Table 2.

**
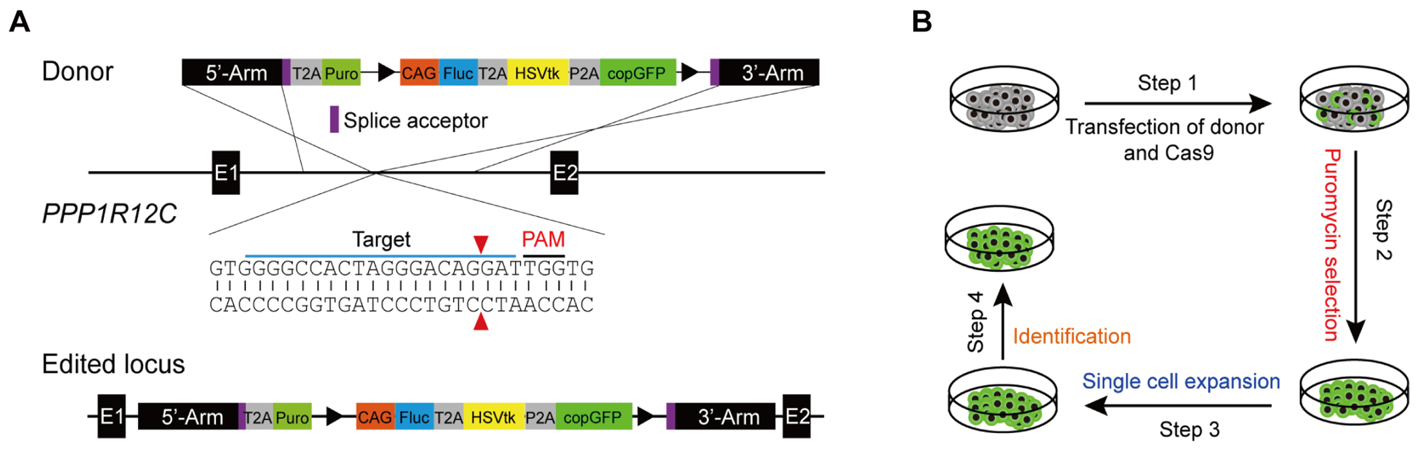
**

**Figure S1.** **Generation of reporter-engineered human embryonic stem cells (ESC-Rep).** (A) Schematic diagram of CRISPR/Cas9-mediated homologous recombination in AAVS1 locus of *PPP1R12C* gene. (B) Procedure of ESC-Rep generation.

**
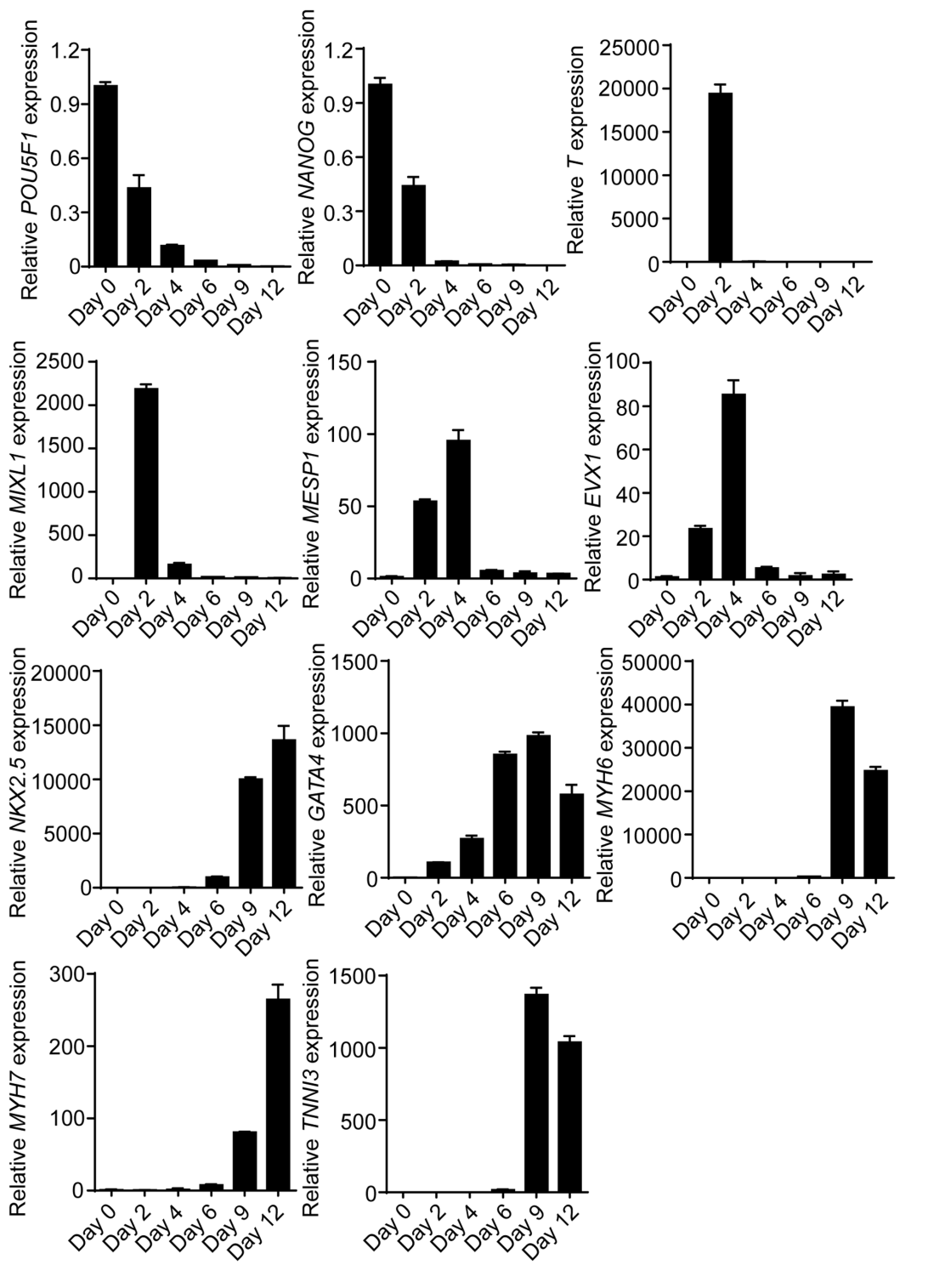
**

**Figure S2. Stage-specific gene expression during cardiomyocyte differentiation.** Real-time PCR analysis for markers of pluripotency (*POU5F1* and *NANOG*), mesoderm (*T* and *MIXL1*), cardiac mesoderm (*MESP1* and *EVX1*), cardiac progenitors (*NKX2.5* and *GATA4*), and cardiomyocytes (*MYH6, MYH7* and *TNNI3*) during the cardiac differentiation.


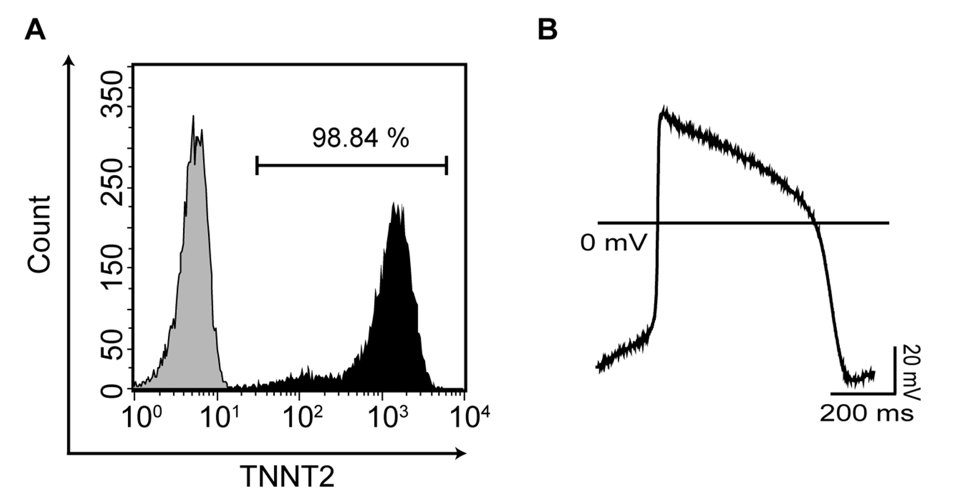


**Figure S3.** **Characteristics of ESC-Rep-CMs**. (A) Flow cytometry assay for troponin T (TNNT2) positive cardiomyocytes after purification; (B) Patch clamp for electrophysiological characteristics of ESC-Rep-CMs.


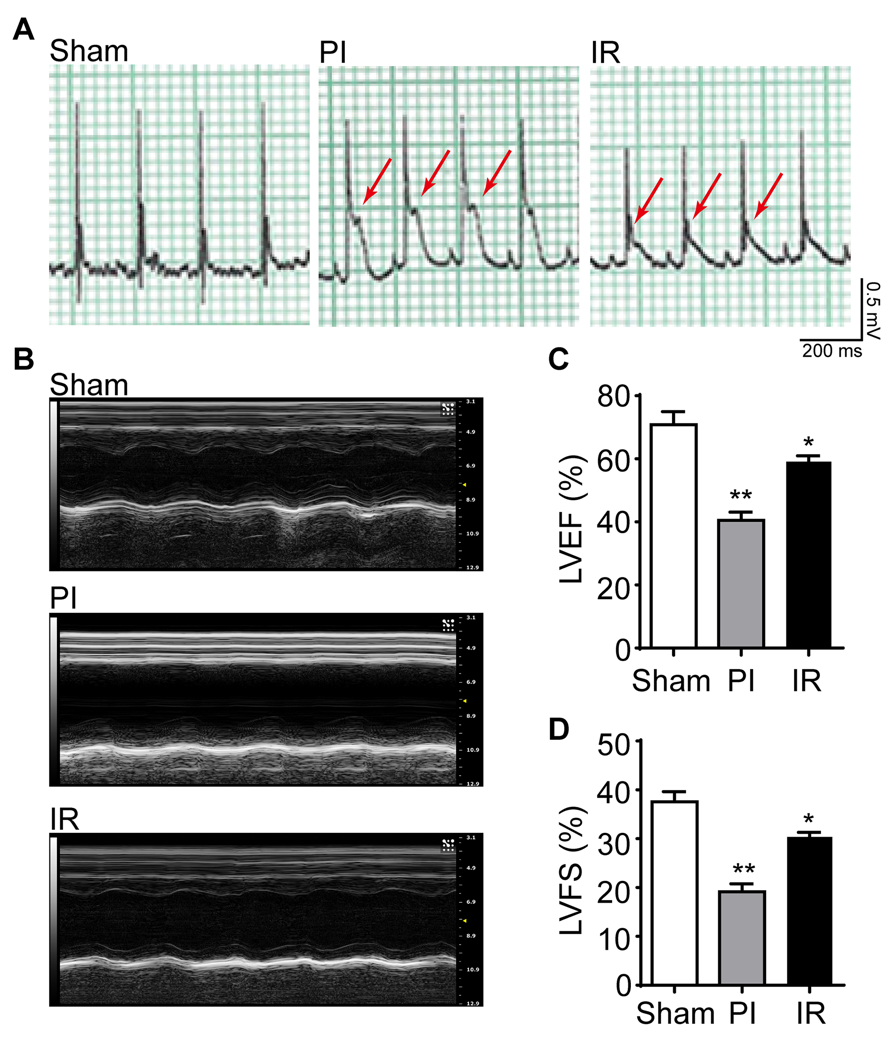


**Figure S4. Post-operative cardiac evaluation in mice.** (A) Electrocardiogram analysis of mice in the Sham, PI and IR groups after surgery. (B-D) Heart function analysis of mice in the Sham, PI and IR groups at day 7 post surgery. All data are presented as the mean ± SEM; one-way ANOVA; **p*<0.05, ***p*<0.01.


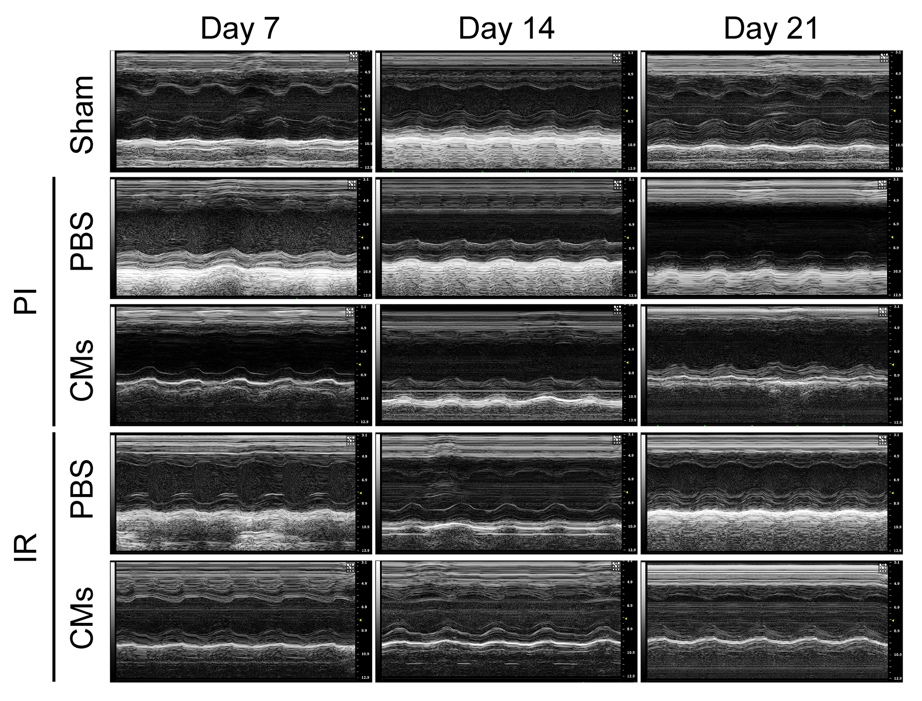


**Figure S5. Representative echocardiogram at Day 7, Day 14 and Day 21.** The representative echocardiogram of mice at Day 7, Day 14 and Day 21 in the Sham, PI group after PBS or ESC-Rep-CM injection, and the IR group after the injection of PBS or ESC-Rep-CMs.


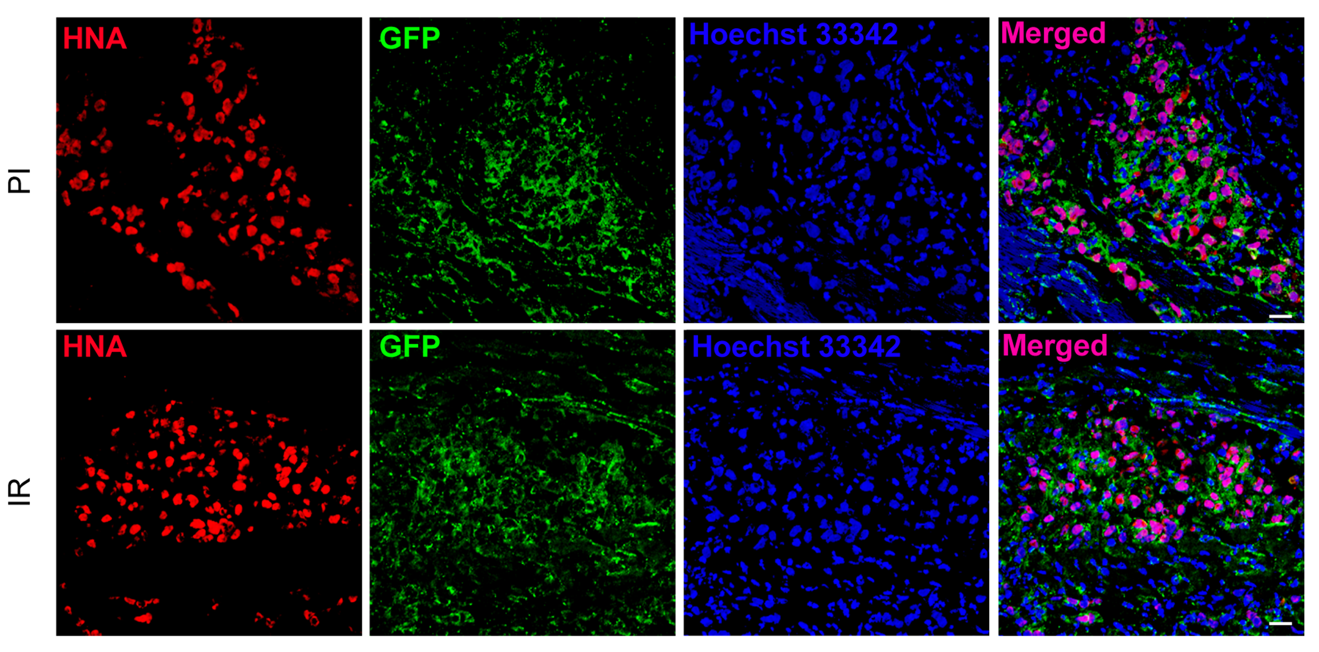


**Figure S6. ESC-Rep-CMs engrafted in mouse infarcted hearts.** Immunostaining of heart sections from the PI and IR groups after ESC-Rep-CM injection for human nuclear antigen (HNA, red), reporter gene GFP (Green) and nuclei (Hoechst 33342, blue). Scale bar, 20 μm.


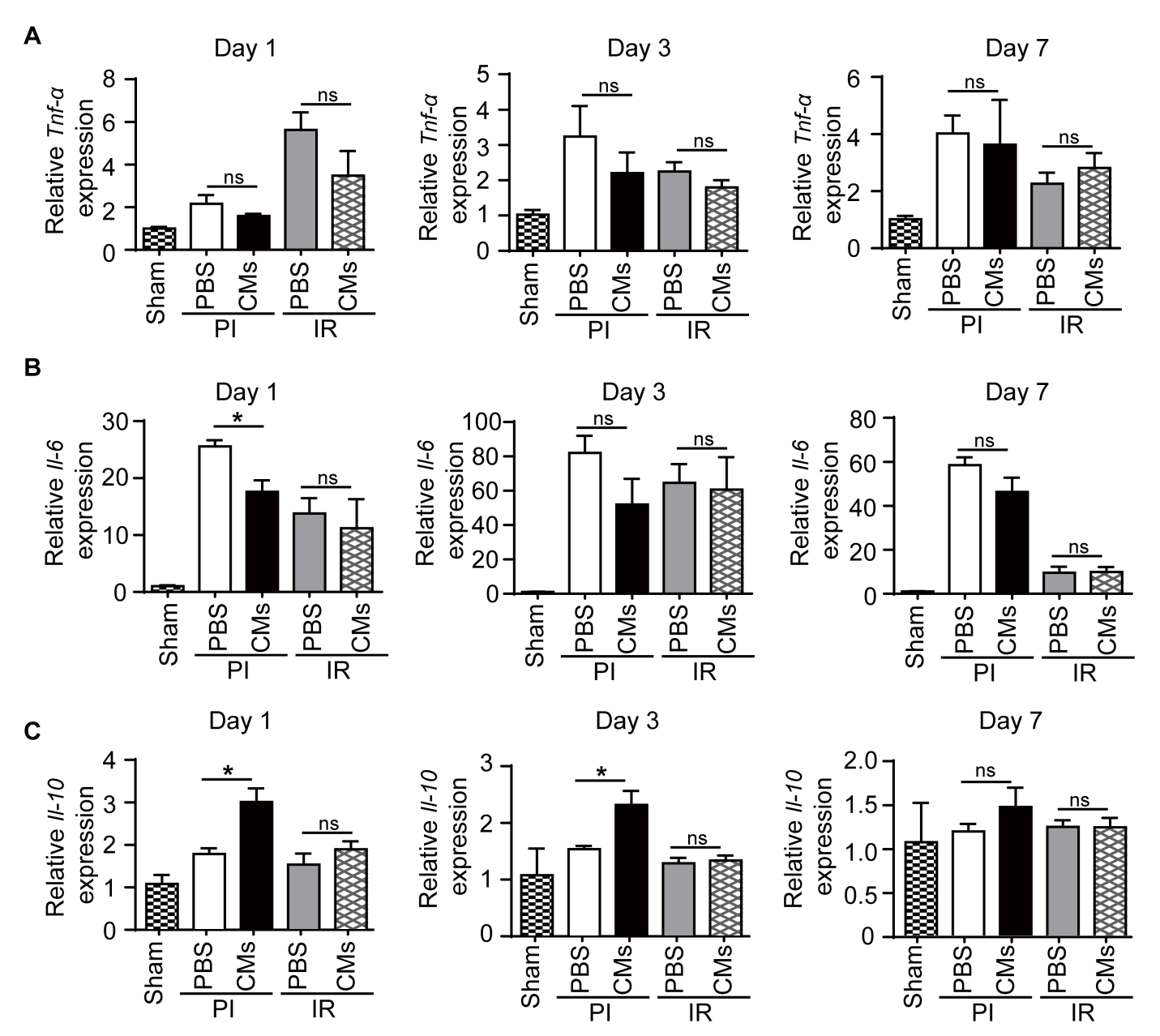


**Figure S7. mRNA expression of inflammatory factors.** (A) Real-time PCR analysis of *TNF-α* at Day 1, Day 3 and Day 7 after cell transplantation. (B) Real-time PCR analysis of *Il-6* at Day 1, Day 3 and Day 7 after cell transplantation. (C) Real-time PCR analysis of *Il-10* at Day 1, Day 3 and Day 7 after cell transplantation. All data are presented as the mean ± SEM; one-way ANOVA; **p*<0.05, and ns, not significant.

**
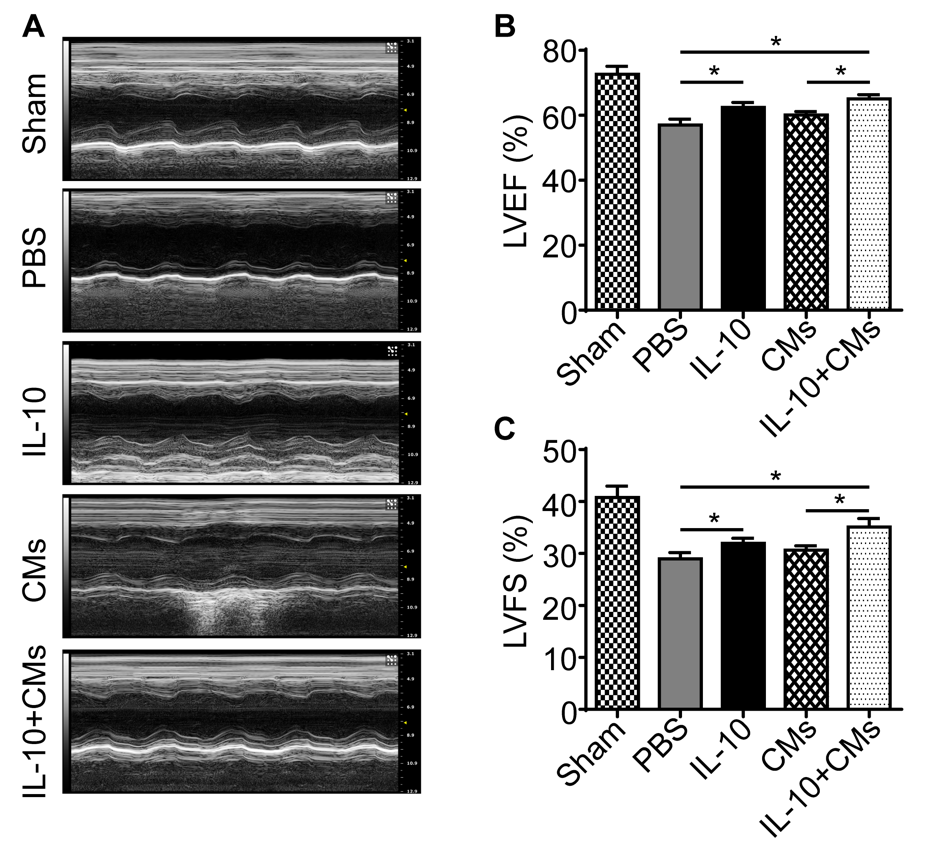
**

**Figure S8. IL-10 mildly improved heart function in IR model.** (A) The representative echocardiogram of mice at Day 7 in the Sham and IR groups accepted PBS, CMs, IL-10 and IL-10+CMs injection, respectively. (B) Quantitative analysis of LVEF in IR mice after indicated treatments. (C) Quantitative analysis of LVFS in IR mice after indicated treatments. *n* = 5 for each group. All data are presented as the mean ± SEM; one-way ANOVA; **p*<0.05.

**Table S1. Primers used in this study.**

| **Gene** | **Forward (5’-3’)** | **Reverse (5’-3’)** |
| --- | --- | --- |
| *GFP* | CTACCACTTCGGCACCTACC | CTCGTACTTCTCGATGCGGG |
| *Fluc* | ACTGGGACGAAGACGAACAC | GGGTGTTGGAGCAAGATGGA |
| *HSVtk* | GGAGGACAGACACATCGACC | ACCGTATTGGCAAGTAGCCC |
| *POU5F1* | CCTCACTTCACTGCACTGTA | CAGGTTTTCTTTCCCTAGCT |
| *SOX2* | CCCAGCAGACTTCACATGT | CCTCCCATTTCCCTCGTTTT |
| *NANOG* | TGAACCTCAGCTACAAACAG | TGGTGGTAGGAAGAGTAAAG |
| *T* | AGATGAACCCAACTGTGGAGAT | GGTGGAGTTTACAAATTCTGG |
| *MIXL1* | CTGCTGGAGCTCGTCTTCC | GGATCCTGGACTCGGGGAG |
| *FOXA2* | AGGGCCAGAGTTCCACAAATC | GGGTATCCCTCCCTCCTTCTT |
| *GATA6* | ACCACCTTATGGCGCAGAAA | ATAGCAAGTGGTCTGGGCAC |
| *SOX1* | GGCTTTTGTACAGACGTTCCC | AACCCAAGTCTGGTGTCAGC |
| *PAX6* | TGGCTCACCAAGGCGAAATA | CCGAGCAGTTGAGTCATTCAG |
| *MESP1* | CCTGAAGGGCAGGCGATG | CCTTGTCACTTGGGCTCCTC |
| *EVX1* | GAAGAAATCGAGGGTCCGGC | GGTAACGACGCATCTGGTCA |
| *NKX2.5* | GCCGCCAACAACAACTTCG | CCTACCAGGCTCGGATACCAT |
| *GATA4* | CTCCTTCAGGCAGTGAGAGC | GCCCGTAGTGAGATGACAGG |
| *TNNI3* | CCCTCACTGACCCTCCAAAC | GAGGTTCCCTAGCCGCATC |
| *MYH6* | AAGCCAACACCAACCTGTCC | TTTGCTTGGCACCAATGTCAC |
| *MYH7* | ACCTGTCCAAGTTCCGCAAG | TCATTCAAGCCCTTCGTGCC |
| *GAPDH* | GAAATCCCATCACCATCTTCCAGG | GAGCCCCAGCCTTCTCCATG |
| *α-SMA* | CCCCCTCTAGTGGTCAGGAA | ACGCTCTCAAATACCCCGTT |
| *Col1a1* | CCGAACCCCAAGGAAAAGA | GTGGACATTAGGCGCAGGA |
| *Col1a2* | AGGAAAGAGAGGGTCTCCCG | GCCAGGAGGACCCATTACAC |
| *Cxcr1* | CCAGCTGGTGCCTCAGATCAAA | GGGCAGCATTCCCGTGAT |
| *Cxcr2* | GCTGCCTACCTTAGGTGTCC | CAGGATACGCAGTACGACCC |
| *Il-10* | GGCCCAGAAATCAAGGAGCA | AATCGATGACAGCGCCTCAG |
| *Il-6* | TCTTCAACCAAGAGATAAGCTGGA | CGCACTAGGTTTGCCGAGTA |
| *Tnf-a* | CGGGCAGGTCTACTTTGGAG | ACCCTGAGCCATAATCCCCT |
| *Ctgf* | AGAACTGTGTACGGAGCGTG | GTGCACCATCTTTGGCAGTG |

**Table S2. Antibodies used in this study.**

| **Antibody** | **Company** | **Cat No** | **Dilution** |
| --- | --- | --- | --- |
| NANOG | Santa Cruz | sc-33759 | 1:200 |
| POU5F1 | Santa Cruz | sc-8628 | 1:200 |
| troponin T | Thermo Scientific | MS-295-P1 | 1:200 |
| Sarcomeric Alpha Actinin | Abcam | ab9465 | 1:200 |
| troponin I | Santa Cruz | sc-8118 | 1:200 |
| TurboGFP Polyclonal | Invitrogen | PA5-22688 | 1:200 |
| Human Nuclei Antigen | Abcam | ab191181 | 1:200 |
| Collagen Type I antibody | Proteintech | 14695-1-AP | 1:1000 |
| GAPDH | Sungene Biotech | KM9002T | 1:1000 |
| Alexa Fluor® 488 AffiniPure Donkey Anti-Rabbit IgG | Jackson ImmuoResearch | 711-545-152 | 1:500 |
| Alexa Fluor® 488 AffiniPure Donkey Anti-Mouse IgG (H+L) | Jackson ImmuoResearch | 715-545-151 | 1:500 |
| Alexa Fluor® 488 AffiniPure Donkey Anti-Goat IgG (H+L) | Jackson ImmuoResearch | 705-545-147 | 1:500 |
| Alexa Fluor® 594 AffiniPure Donkey Anti-Mouse IgG (H+L) | Jackson ImmuoResearch | 715-585-150 | 1:500 |
| Alexa Fluor® 647 AffiniPure Donkey Anti-Rabbit IgG (H+L) | Jackson ImmuoResearch | 711-605-152 | 1:500 |

**Table S3. Cardiac parameters acquired from echocardiography at Day 28**

| **Items** | **Sham(n=6)** | **PI+PBS (n=6)** | **PI+CMs (n=6)** | **P value**  **(PI+PBS *vs* PI+CMs)** | **IR+PBS (n=6)** | **IR+CMs (n=6)** | | **P value**  **(IR+PBS *vs* IR+CMs)** |
| --- | --- | --- | --- | --- | --- | --- | --- | --- |
| LVEF（%） | 72.879±0.373 | 27.974±0.804 | 43.446±0.900 | **0.001** | 58.745±0.774 | 63.650±0.734 | 0.090 | |
| LVFS（%） | 38.725±0.216 | 13.549±0.391 | 21.194±0.455 | **0.001** | 25.815±0.300 | 33.006±0.511 | 0.062 | |
| LV Mass (mg) | 116.625±2.970 | 118.738±3.287 | 111.787±1.938 | 0.418 | 113.721±2.736 | 121.109±3.201 | 0.568 | |
| LV Vol;d (mm^3^) | 49.742±1.161 | 61.093±2.232 | 50.936±1.617 | 0.112 | 50.701±2.190 | 49.487±1.248 | 0.781 | |
| LV Vol;s (mm^3^) | 21.479±0.476 | 29.579±0.603 | 21.794±0.941 | 0.092 | 23.715±1.172 | 22.555±0.630 | 0.769 | |
| E (mm/s) | 669.228±39.248 | 489.679±8.837 | 589.868±9.153 | **0.040** | 630.986±15.351 | 627.799±12.058 | 0.838 | |
| A (mm/s) | 399.45±25.373 | 405.146±9.264 | 388.208±9.962 | 0.548 | 364.141±8.592 | 368.176±8.122 | 0.285 | |
| E/A | 1.801±0.069 | 1.218±0.022 | 1.532±0.021 | **0.008** | 1.732±0.006 | 1.710±0.017 | 0.522 | |

*p*<0.05 was considered as statistically significant.
